# Eomes is sufficient to regulate IL-10 expression and cytotoxic effector molecules in murine CD4^+^ T cells

**DOI:** 10.3389/fimmu.2023.1058267

**Published:** 2023-01-19

**Authors:** Benedikt Thelen, Vincent Schipperges, Paulina Knörlein, Jonas F. Hummel, Frederic Arnold, Laurence Kupferschmid, Christoph S. N. Klose, Sebastian J. Arnold, Melanie Boerries, Yakup Tanriver

**Affiliations:** ^1^ Institute of Medical Microbiology and Hygiene, Faculty of Medicine, University of Freiburg, Freiburg, Germany; ^2^ Institute of Medical Bioinformatics and Systems Medicine, Faculty of Medicine, Medical Center - University of Freiburg, University of Freiburg, Freiburg, Germany; ^3^ Department of Internal Medicine IV, Faculty of Medicine, Medical Center - University of Freiburg, University of Freiburg, Freiburg, Germany; ^4^ Berta-Ottenstein-Programme, Faculty of Medicine, University of Freiburg, Freiburg, Germany; ^5^ Department of Microbiology, Infectious Diseases and Immunology, Charité – Universitätsmedizin Berlin, corporate member of Freie Universität Berlin and Humboldt-Universität zu Berlin, Berlin, Germany; ^6^ Institute of Experimental and Clinical Pharmacology and Toxicology, Faculty of Medicine, University of Freiburg, Freiburg, Germany; ^7^ CIBSS - Centre for Integrative Biological Signalling Studies, University of Freiburg, Freiburg, Germany; ^8^ German Cancer Consortium (Deutsches Konsortium für Translationale Krebsforschung, DKTK), Partner Site Freiburg, and German Cancer Research Center (Deutsches Krebsforschungszentrum, DKFZ), Heidelberg, Germany

**Keywords:** CD4 T cell, Eomes, IL-10, Tr1, LCMV (lymphocytic choriomeningitis virus), cytotoxcicity

## Abstract

The T-box transcription factors T-bet and Eomesodermin regulate type 1 immune responses in innate and adaptive lymphocytes. T-bet is widely expressed in the immune system but was initially identified as the lineage-specifying transcription factor of Th1 CD4^+^ T cells, where it governs expression of the signature cytokine IFN- γ and represses alternative cell fates like Th2 and Th17. T-bet’s paralog Eomes is less abundantly expressed and Eomes^+^ CD4^+^ T cells are mostly found in the context of persistent antigen exposure, like bone marrow transplantation, chronic infection or inflammation as well as malignant disorders. However, it has remained unresolved whether Eomes executes similar transcriptional activities as T-bet in CD4^+^ T cells. Here we use a novel genetic approach to show that Eomes expression in CD4^+^ T cells drives a distinct transcriptional program that shows only partial overlap with T-bet. We found that Eomes is sufficient to induce the expression of the immunoregulatory cytokine IL-10 and, together with T-bet, promotes a cytotoxic effector profile, including *Prf1*, *Gzmb*, *Gzmk*, *Nkg7* and *Ccl5*, while repressing alternative cell fates. Our results demonstrate that Eomes^+^ CD4^+^ T cells, which are often found in the context of chronic antigen stimulation, are likely to be a unique CD4^+^ T cell subset that limits inflammation and immunopathology as well as eliminates antigen-presenting and malignant cells.

## Introduction

T-bet and Eomesodermin (Eomes) are the only members of the diverse family of T-box transcription factors (TF) that play an intrinsic role in immune cell development and function. These two TFs both belong to the Tbr1 group of T-box TFs, and thus display a high grade of homology in their DNA-binding domain, which is called the T-box (1). Nevertheless, their functions only partially overlap and even oppose each other as shown for the activation and differentiation of CD8^+^ T cells during acute and viral infection as well as tumor development (2, 3). T-bet is encoded by *Tbx21* and its role has first been elucidated in CD4^+^ T cells, where it governs Th1 differentiation (1). In contrast, Eomes is scarcely expressed in CD4^+^ T cells and is best known for its role in cytotoxic lymphocytes, i.e. conventional natural killer (NK) cells and activated CD8^+^ T cells, where it enhances effector function and memory formation. While naïve and even classical subsets of CD4^+^ T cells, like Th1, Th2, Th17 or regulatory T cells (Treg), do not express Eomes at high levels, conditions that are associated with chronic or prolonged inflammation can induce Eomes-expressing CD4^+^ T cells. While the role of its paralogue T-bet in CD4^+^ T cells is well understood, the precise functions of Eomes in such CD4^+^ T cells remain unclear (4).

In several human diseases and associated mouse models, Eomes is expressed in CD4^+^ T cells, but its overall role is still unresolved, as Eomes^+^ CD4 T cells show aspects of pro- and anti-inflammation. On a cellular level Eomes guides differentiation, cytokine production, and promotion of cytotoxicity (5). These functions can lead to inflammation and, when dysregulated, to autoimmunity. For example, patients with secondary progressive multiple sclerosis show elevated numbers of Eomes expressing CD4^+^ T cells that mark inflammatory disease activity (6). Furthermore, in a mouse model for experimental autoimmune encephalitis (EAE), Raveney et al. showed that deletion of Eomes suppresses chronic EAE (7). Mazzoni et al. reported that Eomes^+^ CD4^+^ T cells are also enriched in synovial fluid of patients with juvenile idiopathic arthritis (8).

In contrast to the aforementioned, Eomes has been associated with differentiation of immune regulatory Tr1 CD4^+^ T cells (9–11) that are independent of the TF FoxP3 and produce large amounts of the anti-inflammatory cytokine IL-10. Zhang et al. showed that Tr1 CD4^+^ T cells are abundant after bone marrow transplantation in mice and humans, and that Eomes is essential for the immunosuppressive functions limiting graft versus host disease in mice (9). Underlining a potential immune regulatory role, reduced numbers of Eomes^+^ CD4^+^ T cells are found in inflamed tissue of inflammatory bowel disease patients (10). Finally, high numbers of Eomes^+^ Tr1-like CD4^+^ T cells in colorectal cancer and non-small-cell lung cancer correlate with disease progression and metastasis (12). Besides anti-inflammatory functions, Tr1-like CD4^+^ T cells potentially also control leukaemia by harnessing cytotoxic effector functions that are regulated by Eomes. Roessner et al. found that cytotoxic, Eomes^+^ CD4^+^ T cells were enriched in lymph nodes of chronic lymphocytic leukaemia patients, as well as in a corresponding mouse model, Eµ-TCL1. While Eomes^+^ CD4^+^ T cells co-transferred with Eµ-TCL1 cells in *Rag2^-/-^* mice suppressed leukaemia, Eomes deficient cells failed to do so. Finally, a comparative transcriptomic analysis of Eomes-sufficient and -insufficient CD4^+^ T cells revealed that Eomes was linked to a Tr1 cell-specific gene signature (11). Yet it remains to be determined whether anti-inflammatory IL-10 production and cytotoxic effector function is executed by the same cell or whether different subsets exist, that simply utilize different aspects of Eomes’ transcriptional activity but are otherwise transcriptionally distinct.

The numerous associations of Eomes to states of chronic inflammation clearly demand an exact outlining of its role in CD4^+^ T cells. So far Eomes expression in CD4^+^ T cells has predominantly been evaluated in disease models under preselected conditions, which often result in a substantial heterogeneity of CD4^+^ T cell subsets (13). These conditions may not only favour Eomes expression, but also select other transcriptional regulators altering the differentiation and effector functions of CD4^+^ T cells. Considering the diverse roles suggested for Eomes it remains to be clarified whether Eomes is sufficient to induce the described phenotypes. This study thus investigates the role of Eomes in CD4^+^ T cells in a novel mouse model that allows for the titrated and activation-induced expression of Eomes in CD4^+^ T cells. *Via* phenotypical and functional *in vitro* and *in vivo* analysis as well as transcriptional profiling, we show that Eomes regulates a distinct phenotypical and transcriptional program in CD4^+^ T cells. While some functions overlap with T-bet, with both factors promoting Th1 features like IFN-γ, only Eomes induces a Tr1-like program including production of IL-10 and the expression of cytotoxic effector molecules.

## Materials and methods

### Mice

*Tbx21* ^+^ *^/E^*, *Tbx21* ^+^ *^/-^* and SMARTA TCR transgenic (tg) mice on C57BL/6 background were crossed and bred locally. Mice of same sex between 8 to 12 weeks of age and, if possible, littermates were used. Female C57BL/6 mice at age of seven weeks were purchased from Janvier Laboratories LeGenest St-Isle, France) for transfer experiments. All experiments were performed according to the local animal care committees of the University of Freiburg and the Regierungspräsidium Freiburg.

### *In vitro* anti-CD3 stimulation assay

Single cell suspensions of 1.5x10^5^ splenocytes were incubated with 1 μg/ml anti-CD3 (145-2C11; BioLegend; San Diego, CA, US) with or without 50 ng/ml IL-12 (PeproTech; Hamburg, Germany) in 200 μl complete medium (RPMI 1640; supplemented with 1% Sodium Pyruvate, 0.1% 2-Mercaptoethanol, 1% L-Glutamine, 1% Penicillin/Streptomycin; all from Life Technologies; 10% FCS; PAN Biotech, Aidenbach, Germany; 10 mM Hepes Sigma-Aldrich) in u-bottom 96-well plates (Sigma-Aldrich; St.Louis, MO, US) for 48 or 72 hours.

### *In vitro* GP (61–80) stimulation assay

Single cell suspensions of 1.5x10^5^ SMARTA-splenocytes were incubated with 1x10^-8^ M LCMV GP (61-80) (AS-64851; Eurogentec; Seraing; Belgium) with 50 ng/ml IL-12 (PeproTech; Hamburg, Germany) in 200 μl complete medium (RPMI 1640; supplemented with 1% Sodium Pyruvate, 0.1% 2-Mercaptoethanol, 1% L-Glutamine, 1% Penicillin/Streptomycin; all from Life Technologies; 10% FCS; PAN Biotech, Aidenbach, Germany; 10 mM Hepes Sigma-Aldrich) in u-bottom 96-well plates (Sigma-Aldrich; St.Louis, MO, US) for 72 hours.

### Adoptive transfer and infection

SMARTA CD4^+^ T cells express a transgenic (tg) TCR specific for the H2-I-A^b^ restricted LCMV GP_61-80_ epitope. From spleens of SMARTA TCR tg *Tbx21^+/+^*, *Tbx21^+/-^*, *Tbx21^+/E^* and *Tbx21^-/E^* mice on a CD90.1 (Thy1.1) background SMARTA CD4^+^ T cells were isolated by Dynabeads Untouched Mouse CD4 Cells Kit (Invitrogen, ThermoFisher Scientific; Waltham, MA, US) with a purity of > 97%. For each genotype 2x10^4^ SMARTA (CD90.1^+^) CD4^+^ T cells were intravenously injected into five C57BL/6 recipient mice on a CD90.2 background. One day later mice were infected intravenously with 200 plaques forming units LCMV WE. On day eight after infection spleens were harvested for further analysis of SMARTA CD4^+^ T cells. For the correlation of weight gain between the groups mice were kept for 30 days after infection and weight measured two times per week.

### Immunofluorescence microscopy

Eight days after adoptive transfer and infection one third of the spleens was fixated in 4% formaldehyde for two hours, dehydrated in 30% sucrose for 24 hours and frozen with liquid nitrogen in Tissue-tek O.C.T. compound (Sakura Finetek Europe; Alphen aan den Rijn, Netherlands). On glass slides cryostat sections of 7 μm thickness were blocked with 5% BSA (Sigma-Aldrich) for 30 minutes, followed by staining with PE conjugated CD90.1 antibody diluted 1:500, FITC conjugated CD45R/B220 antibody and APC conjugated CD4 antibody both diluted 1:200 in 5% BSA, containing DAPI (all from BioLegend) diluted 1:8000. After one hour the slides were washed with PBS three times for three minutes and mounted with mounting medium (PermaFlour by Thermo Scientific, ThermoFisher). Images were taken at an Axioplan 2 fluorescence microscope at 20x magnification using an AxioCam camera and Axiovision LE software Rel. 4.9 (all Axio devices were from Zeiss, Oberkochen, Germany). Stitching as well as computational cell detection and counting were performed in QuPath-0.2.3 (University of Edinburgh). Single cells were identified by DAPI signal and B cells, CD4 T cells and SMARTA CD4 T cells by their respective fluorescence coupled antibody (FITC/B220, APC/CD4 and PE/CD90.1). B cell zones were marked manually, and cell numbers counted computationally.

### RNA isolation, cDNA synthesis and real-time quantitative PCR

RNA was extracted by using TRIzol Reagent (Life Technologies, ThermoFisher). Quantity and purity were measured with a NaNodrop 8000 (ThermoScientific). An adsorption quotient of > 1.7 of the wavelengths 260/280 was accepted. 200 ng of RNA was used for reverse transcription with the High Capacity cDNA Reverse Transcription Kit. Quantitative Real-Time qPCR was performed with the SYBR Green Master Mix on a QuantStudio 5 (all ThermoFisher), with the following primer pairs: *Tbx21* (fw 5´-ATCGACAACAACCCCTTTGC-3´; rev 3´-GACACTCGTATCAACAGATGCG-5´), *Eomes* (fw 5´-AAATTCCACCGGCACCAAAC-3´; rev: 3´-CCAGAACCACTTCCACGAAAAC-5´), *Gzma* (fw 5´- CGATGAGGAACGCCTCTGGT-3´; rev: 3´- AGCGCCAGCACAGATGGTAT-5´), *Gzmb* (fw 5´- GCGATATGTGGGGGCTTCCT-3´; rev: 3´- GTGGGCCCCCAAAGTGACAT-5´), *Prf1* (fw 5´- CTGGCTCCCACTCCAAGGTA-3´; rev: 3´- AGCTGTTAAAGTTGCGGGGG-5´), *Nkg7* (fw 5´- TGGCCCTCTGGTCTCAACTGT-3´; rev: 3´- AGTGAGCACCCAGGCTCAGG-5´), *Il10ra* (fw 5´- ATTGCATACGGGACAGAACTGC-3´; rev: 3´- ACAGGACAATGCCTGAGCCT-5´), *Il10* (fw 5´- TAGAAGTGATGCCCCAGGCAG-3´; rev: 3´- TAGACACCTTGGTCTTGGAGC-5´). For calculation of the relative expression, ct-values were normalized with *Gapdh* (fw 5´-GGAGCGAGATCCCTCCAAAAT-3´, rev 3´-GGCTGTTGTCATACTTCTCATGG-5´) or *Hprt* (fw 5´-TGATCAGTCAACGGGGGACA-3´, rev 3´-TTCGAGAGGTCCTTTTCACCA-5´).

### Flow cytometry and cell sorting

In line with the published guidelines flow cytometry was performed with antibodies obtained from BioLegend, Thermo Fisher Scientific and BD Bioscience (San Jose, CA, USA). Surface staining was performed in PBS for 30 minutes with anti-CD16/CD32 antibody for FC receptor blocking and the following antibodies: CD4 (RM4-5), CD8a (53-6.7), CD44 (IM7), CD127 (A7R34), CD122 (TM-beta1), CD27(LG.3A10), CD39 (Duha59), Ccr5 (HM-CCR5), CD90.1 (OX-7), CD62L (MEL-14), Ly6c (HK1.4), PSGL-1 (2PH1), TIM-3 (RMT3-23), LAG3 (C9B7W), CXCR3 (CXCR3-173), PD-1 (29F.1A12). Viability staining was performed with BD Horizon Fixable viability stain 510 (BD Bioscience) diluted 1:1000 in PBS for 30 minutes. Intranuclear staining was performed with FoxP3 transcription factor staining buffer set (Thermo Fisher Scientific) according to manufacturer instructions with the following antibodies: T-bet (eBio4B10), Eomes (Dan11mag). For intracellular staining 2x10^6^ cells were incubated with 0,5 µg/ml Ionomycin, 25 ng/ml PMA (both Sigma-Aldrich) and 10 µg/ml Brefeldin A (BioLegend) in 200 µl RPMI in u-bottom 96 well plates for four hours at 37° C with 5% CO_2_. After surface staining and viability staining cells were fixated in 60 µl BD Cytofix/Cytoperm (BD Bioscience) for 20 minutes. Incubation with the following antibodies was performed in 0,5% saponine/PBS: IFN-γ (XMG1.2), TNF (MP6-XT22), IL-10 (JES5-16E3), IL-17A (TC11-18H10.1), GzmB (QA18A28), IL-2 (JES6-5H4). Acquisition was performed with a LSRFortessa (BD Bioscience) flow cytometer and analysed using FlowJo software V10.7.1 (Treestar; Ashland, Oregon, US). Cell sorting was performed at a FACS Aria Fusion or FACS Aria III (BD Bioscience) at the Lighthouse Core Facility of the Universitätsklinikum Freiburg, Germany.

### RNA isolation, cDNA synthesis and RNA sequencing

RNA was extracted by using RNeasy Micro Kit (Quiagen; Hilden, Germany) and treated with DNase I to eliminate possible genomic DNA contamination. Quantity and Purity were measured with High Sensitivity RNA ScreenTape (Agilent; Santa Clara, CA, USA). cDNA synthesis, amplification and library preparation were performed with NEBNext Single Cell/Low Input RNA Library Prep Kit (New England Biolabs Gmbh; Frankfurt, Germany). Sequencing was performed on a HiSeq 4000 (Illumina; San Diego, CA, USA), with 58 million reads per sample.

### RNA-seq analyses

The raw RNA sequencing files were pre-processed using Trimmomatic (14), clipping adapters removing reads with low base quality (< 20). The trimmed reads were then aligned using STAR (15) with the GRCm38 mouse reference genome from Ensembl. Normalization and differential gene expression analysis was performed with the R/Bioconductor (16) package DESeq2 (17) separately for each of the six pairwise comparisons between the four groups. Genes with a Benjamini-Hochberg adjusted p-value below 0.05 were considered significant in the respective comparison. The Principal Component Analysis (PCA) was performed using the FactoMineR package. Upset plots and heatmaps were generated with the packages UpSet (18) and pheatmap (RRID : SCR_016418) respectively.

### Gene set enrichment analysis

Enrichment analysis was performed using the R/Bioconductor package GAGE (Generally Applicable Gene Set Analysis) (18) with pathways from the KEGG Database (19). Pathways were considered significant with a Benjamini-Hochberg adjusted p-value below 0.05. For comparison with human Tr1 cells, we used the list of genes published by Gruarin et al. that were classified as “IL7R- IL10+ UP” (10). These genes were linked to their homologous mouse genes, using the Mouse Genome Informatics Database (extracted December 2022). The separate gene lists for Th1, Th2, Th17 and Treg cells are taken from Stubbington et al. (20), where each gene is associated with a cell type in which it is upregulated most.

### Statistics

If not otherwise indicated one-way ANOVA with Tukey´s post-test for multiple comparison was performed with Graph Pad Prism 8.4.3 (Graph Pad Software; La Jolla, CA, USA). [p < 0.05(*), p < 0.01(**), p < 0.01(***); not significant (ns)].

### Data and software availability

RNA sequencing (RNA-seq) data sets are available at NCBI GEO (GSE213980).

## Results

### *Tbx21^+/E^* mice co-express T-bet and Eomes in CD4^+^ T cells

**Figure 1:**
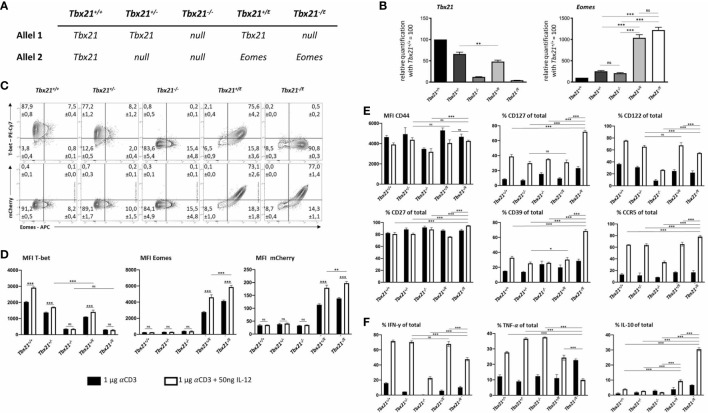
Eomes induces IL-10 production in CD4^+^ T cells *in vitro. In vitro* stimulation of splenocytes with soluble Anti-CD3 with or without IL-12 for 48 hours, cells were analyzed by RT-qPCR **(B)** or flow cytometry **(C-F)**. **(A)** Table of Tbx21 alleles corresponding to genotypes with *+* = *Tbx21* (encoding for T-bet), E = *Eomes*, - = null allele. **(B)** RT-qPCR of *Tbx21* and *Eomes* in relation to house-keeping genes GAPDH and HPRT after stimulation with Anti-CD3 and IL-12. Indicated genotypes were further normalized to *Tbx21^+/+^* (wild type), set as 100. **(C)** Intranuclear staining of T-bet and Eomes after stimulation with anti-CD3 and IL-12. Representative Dot plots are shown. **(D)** Mean fluorescence intensities (MFI) of T-bet, Eomes and mCherry. **(E)** Surface staining for CD44 and intracellular staining **(F)** for TNF, IFN-γ and IL-10 after 4 hours of stimulation with PMA, Ionomycin and Brefeldin **(A)** Statistical analysis: *=p < 0.05; **=p < 0.01; ***=p < 0.001; ns, not significant; Error bars denote mean + SEM. n=3.

Our group previously described a new mouse allele, *Tbx21^E^*, which results in the expression of Eomes in combination with the fluorescence reporter mCherry *in lieu* of T-bet, while also creating a *Tbx21* null allele (21). We now used this allele in combination with previously generated *Tbx21^-^* (knockout allele) and the *Tbx21^+^* wild type allele to establish five different mouse lines with titrated expression of T-box transcription factors from the *Tbx21* locus (**Figure 1A**). The *Tbx21 locus* is readily activated in CD4^+^ T cells under Th1 polarising conditions. This in turn uncouples the induction of Eomes from the *Tbx21^E^* allele from specific environmental signals, which thus allows us to gauge the effects of the different T-box TF combinations (**Figure 1A**) on CD4^+^ T cell differentiation. In summary, our well-defined genetic approach minimizes the impact of specific external cues on the induction of T-bet or Eomes but rather focusses on their unique, synergistic and joint down-stream effects. We have previously shown that such a strategy is valid for CD8^+^ T cells and we therefore started our study by determining the faithfulness of our transgenic model in murine CD4^+^ T cells. Hence, splenocytes of the different genotypes were polyclonally activated with anti-CD3 antibody. Furthermore, exogenous IL-12 was added to the wells to promote activation of the *Tbx21* locus through STAT4 (22). Haploinsufficiency for *Tbx21* (*Tbx21^+/+^ versus Tbx21^+/-^* and *Tbx21^+/E^*) showed a reduction of nearly 50% in *Tbx21* transcripts, which implies that T-bet requires bi-allelic expression. Transcriptional profiling for *Tbx21* and *Eomes* mRNA expression clearly confirmed the faithfulness of our *Tbx21^E^* allele, as *Tbx21^-/E^* cells showed no expression of *Tbx21* (**Figure 1B**, left), whereas only *Tbx21^+/E^* and *Tbx21^-/E^* cells showed a relevant and strong expression of *Eomes* (**Figure 1B**, right). Interestingly, we only detected a slight increase of *Eomes* transcripts in *Tbx21^+/-^* and *Tbx21^-/-^* cells (**Figure 1B**, right), arguing that neither haploinsufficiency for *Tbx21* nor its complete absence results in the default and uniform induction of *Eomes* in CD4^+^ T cells. This set of data was corroborated by flow cytometry (**Figures 1C, D**) for T-bet, Eomes and mCherry in activated CD4^+^ T cells. In line with our transcriptional data, *Tbx21* haploinsufficiency resulted in the reduced expression of T-bet, which could only be partially rescued by the addition of IL-12 (**Figures 1C, D**). As predicted, *Tbx21^-/-^* and *Tbx21^-/E^* CD4^+^ T cells showed no expression of T-bet (**Figure 1C**, upper row). Gradual loss of *Tbx21* (*Tbx21^+/+^* >*Tbx21^+/-^* >*Tbx21^-/-^*) showed only a slight but stepwise increase in Eomes expression (**Figures 1C, D**). In contrast, the introduction of our novel *T-bx21^E^* allele resulted in striking and uniform expression of Eomes in CD4^+^ T cells from *Tbx21^+/E^* and *Tbx21^-/E^* mice. The fact that Eomes expression in *Tbx21^+/E^* and *Tbx21^-/E^* cells was paralleled by a fluorescence increase of the mCherry reporter (**Figure 1C, D**) clearly indicates that the observed Eomes expression in CD4^+^ T cells from *Tbx21^+/E^* and *Tbx21^-/E^* mice is mainly driven from the *Tbx21^E^* allele and not the endogenous *Eomes* allele. Finally, addition of IL-12 led to a significant increase of Eomes and mCherry expression, which further supports the conclusion that our novel *Tbx21^E^* allele is under the full transcriptional control of the *Tbx21* locus in CD4^+^ T cells. In summary, these results show that the expression and dynamic regulation of Eomes from the *Tbx21^E^* allele mimics the pattern of T-bet in wild type CD4^+^ T cells.

### Eomes induces Tr1-like features *in vitro*

With the faithful expression of Eomes in CD4^+^ T cells confirmed, we investigated phenotypical differences between the different T-box TF genotypes after polyclonal activation. As T-bet is the lineage-defining TF of Th1 CD4^+^ T cells and Eomes is linked to the development of regulatory Tr1 CD4^+^ T cells, we primarily focussed on these two differentiation pathways. Global activation as assessed by the marker CD44 was similar across all genotypes (**Figure 1E**). It has been shown that expression of the IL-2Rβ chain (CD122) is jointly regulated by T-box TF in CD8^+^ T cells (23). Accordingly, we could see a stepwise loss (*Tbx21^+/+^* >*Tbx21^+/-^* >*Tbx21^-/-^*) which was rescued in CD4^+^ T cells that harboured the *Tbx21^E^* allele (**Figure 1E**, upper row). In contrast, CD127, which can be repressed by T-bet (24) was significantly increased in *Tbx21^-/E^* CD4^+^ T cells. The fact that this effect was not observed in *Tbx21^+/E^* CD4^+^ T cells argues that Eomes cannot override T-bet’s effects here, in case both TF are present (**Figure 1E**). Besides the secretion of IL-10, Tr1 cells are characterized by a distinct combination of surface markers, including CD49b, CD27, CD39 and the chemokine receptor CCR5 (25). We could not detect CD49b in any of our genotypes (not shown) and CD27 did not show any difference. However, we noticed a significant increase of CD39 and CCR5 in CD4^+^ T cells from *Tbx21^-/E^* mice after IL-12 treatment. As Eomes expression is significantly lower without the addition of IL-12 (**Figure 1D**, middle) this might indicate that Eomes expression must reach a certain threshold before it can activate these targets or that it requires additional partners that rely on signalling *via* IL-12. Finally, we explored the cytokine profile of activated CD4^+^ T cells. As predicted, expression of T-bet was important for the induction of IFN-γ and was therefore severely curtailed in *Tbx21^-/-^* CD4^+^ T cells (**Figure 1F**, left). Eomes could compensate for the loss of T-bet as *Tbx21^-/E^* CD4^+^ T cells produced significantly higher levels of IFN-γ as *Tbx21^-/-^* CD4^+^ T cells (**Figure 1F**, left). TNF production was independent of T-bet, but showed significant repression by the joined actions of Eomes and IL-12 (**Figure 1F**, middle). Most interestingly, transgenic expression of Eomes in CD4^+^ T cells from *Tbx21^+/E^* mice led to marked upregulation of IL-10, which was amplified in CD4^+^ T cells from *Tbx21^-/E^* mice and thus in the absence of T-bet (**Figure 1F**, right). In summary, our *in vitro* results support the hypothesis that Eomes expression in CD4^+^ T cells guides a distinct phenotype which shows only partial overlap with the actions of T-bet and bears unique hallmarks of Tr1 CD4^+^ T cells.

### Redundancy of T-bet in CD4^+^ T cells during acute infection with LCMV WE

**Figure 2:**
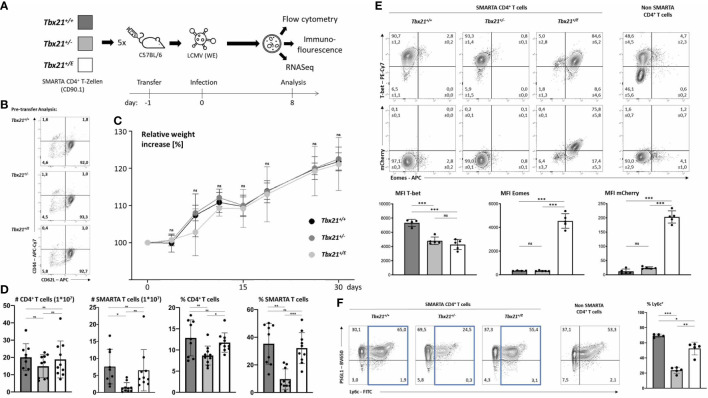
Eomes promotes a distinct phenotype in CD4^+^ T cells during acute infection. **(A)** CD4^+^ T cells were purified and 2x10^4^ cells were transferred into five C57BL/6 mice per genotype. After 24 hours mice were infected with 200 pfu LCMV (WE). On day 8 after infection cells were counted and analysis was performed by flow cytometry, immunofluorescence microscopy or sorting of CD90.1 positive SMARTA CD4^+^ T cells and RNA sequencing. **(B)** Pre-transfer analysis and confirmation that naïve CD4+ T cells (CD62L^+^CD44^-^) were transferred. **(C)** Relative weight increases in relation to date of infection. **(D)** Cell numbers of CD4^+^ T cells (CD4^+^CD90.2^+^) and SMARTA CD4 T cells (CD4^+^CD90.1^+^) were calculated by multiplying absolute cell numbers with the percentages measured by flow cytometry. Dots in the bar diagrams represent individual mice out of two independent experiments. **(E)** Intranuclear staining of T-bet and Eomes shown in dot plots and the MFI in bar diagrams. **(F)** Surface staining of Ly6c and PSGL-1 shown in dot blots and MFI in bar diagrams. SMARTA CD4^+^ T cells were identified as CD4^+^CD90.1^+^. Endogenous non-SMARTA CD4^+^ T cells (CD4^+^CD90.1^-^) were used as a staining control and for setting quadrant gates in **(E, F)**. Statistical analysis: *=p < 0.05; **=p < 0.01; ***=p < 0.001; ns, not significant; Error bars denote mean + SEM. n=2 with 4-5 mice per group. If not otherwise indicated, one representative experiment is shown.

The data so far clearly indicated that Eomes can govern a unique transcriptional program in CD4^+^ T cells. To gain further insight, we next established an acute infection model as we reasoned that the *in vitro* experiments we performed so far could not sufficiently recapitulate all conditions that are present *in vivo*. We chose an acute infection model instead of a chronic one as the latter could induce subsets of Eomes^+^ CD4 T cells even in wild type cells, which would interfere with our downstream analysis (13) by not having a proper negative control. As we were primarily interested in the intrinsic effects of T-box TF and as the *Tbx21* promoter is active in various cell types of the innate and adaptive immune system these experiments could only be done rigorously in an adoptive transfer setting into clonotypic wild type mice. This approach avoided confounding extrinsic effects from bystander lymphocytes (e.g. CD4^+^ T cells, NKT cells, innate lymphoid cells (ILC)) which would otherwise harbor the same T-box TF matrix as the responding CD4 T cells. For this purpose, we crossed clonotypic (CD90.1) T cell receptor (TCR) transgenic SMARTA mice (26), that recognize the H2-I-A^b^ restricted LCMV GP_61-80_ epitope with different T-box TF genotypes to obtain *Tbx21^+/+^*, *Tbx21^+/-^* and *Tbx21^+/E^* on a SMARTA background. We focussed on *Tbx21^+/E^*, and not *Tbx21^-/E^*, as Tr1 cells show dual expression of T-bet and Eomes (9) and used *Tbx21^+/-^* mice as an additional control for *Tbx21* haploinsufficiency as *Tbx21^+/E^* mice harbour only one intact *Tbx21* wild type allele. Next, we adoptively transferred small numbers of SMARTA CD4^+^ T cells from these three genotypes into separate CD90.2 C57BL/6 wild type mice (day -1) and infected them with 200 plaque forming units (pfu) LCMV WE (day 0), which results in an acute infection (**Figure 2A**) that we analysed on day 8. We did not check for viral persistence, as we (21) and others (27) have previously shown that, irrespective of adoptively transferred T cells, C57BL/6 wild type mice can robustly clear an infection with 200 pfu LCMV in the spleen after 8 days. A pre-transfer analysis confirmed the naïve phenotype of SMARTA CD4^+^ T cells independently of the genotype (**Figure 2B**). All three groups showed a similar weight gain over a 30-day period and no overt signs of immunopathology were observed (**Figure 2C**). Splenic SMARTA CD4^+^ T cells from haploinsufficient *Tbx21^+/-^* mice showed a significant reduction in numbers and frequency, which was rescued in CD4^+^ T cells from *Tbx21^+/E^* mice (**Figure 2D**). Hence, Eomes can compensate for the loss of at least one *Tbx21* allele during clonal expansion. Intriguingly, we also detected a partial reduction of endogenous CD4^+^ T cells in mice that were transferred with CD4^+^ T cells from haploinsufficient SMARTA *Tbx21^+/-^* mice. Again, this reduction was rescued after transfer from SMARTA *Tbx21^+/E^* mice, which hints at a cross talk between endogenous and transferred CD4^+^ T cells. In accordance with our *in vitro* data (**Figures 1C, D**) CD4^+^ T cells from SMARTA *Tbx21^+/+^* mice showed the highest expression of T-bet, whereas CD4^+^ T cells from haploinsufficient SMARTA *Tbx21^+/-^* mice and SMARTA *Tbx21^+/E^* mice expressed only half the amount (**Figure 2E**). This extends and corroborates our *in vitro* data and underlines the importance of using SMARTA *Tbx21^+/-^* mice as an additional control in our system. Moreover, only CD4^+^ T cells from SMARTA *Tbx21^+/E^* mice showed a strong and uniform induction of Eomes, which was also not observed in the endogenous CD4^+^ T cell compartment. This Eomes expression was mainly driven from the *Tbx21^E^* allele, as it exquisitely correlated with mCherry fluorescence intensity (**Figure 2D**). Finally, we analysed the expression of PSGL-1 and Ly6C, as Ly6C^high^ CD4^+^ T cells depend on enhanced expression of T-bet for its formation. In combination with Ly6c, PSGL-1 has been proposed as a marker separating a Th1 effector phenotype (Ly6c^+^PSGL-1^+^) from a Th1 memory phenotype (Ly6c^-^PSGL-1^+^) and from a follicular helper T cell (Tfh) enriched population (Ly6c^-^PSGL-1^-^) (28). Supporting this model, CD4^+^ T cells from haploinsufficient SMARTA *Tbx21^+/-^* mice showed the lowest number of Ly6C^+^ T cells, which could, however, be partially rescued in SMARTA *Tbx21^+/E^* CD4^+^ T cells (**Figure 2F**). None of the genotypes showed an enhancement of the Tfh-enriched Ly6c^-^PSGL-1^-^ population. Collectively, our *in vivo* data so far are in line with a model in which Eomes, if timely induced, can in part compensate for the loss of T-bet.

### Eomes induces a Tr1 like phenotype in CD4^+^ T cells

**Figure 3:**
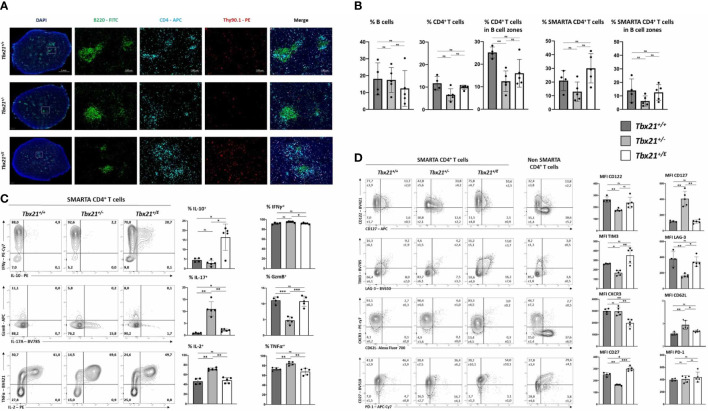
Eomes induces IL-10 production in CD4^+^ T cells but does not alter migration in spleen during acute infection. 2x10^4^ SMARTA CD4^+^ T cells were transferred into C57BL/6 mice, following LCMV infection as shown in **Figure 2A**. **(A)** Immunofluorescence staining with indicated antibodies and DAPI. An overview and single staining for representative B cell zones are shown. **(B)** Relative cell numbers were calculated by computational marking and counting of cells in the total spleen and in marked B cell zones. **(C)** Intracellular staining of cytokines was performed after 4 hours of stimulation with PMA, Ionomycin and Brefeldin **(A)** Representative dot plots and the MFI in bar diagrams are shown. **(D)** Surface staining of indicated markers shown in dot blots and MFI in bar diagrams. SMARTA CD4^+^ T cells were identified as CD4^+^CD90.1^+^. Endogenous non-SMARTA CD4^+^ T cells (CD4^+^CD90.1^-^) were used as a staining control and for setting quadrant gates in **(C, D)**. Statistical analysis: *=p < 0.05; **=p < 0.01; ***=p < 0.001; ns, not significant; Error bars denote mean + SEM. n=2 with 4-5 mice per group.

In response to an acute infection, virus-specific CD4^+^ T cells clonally expand and differentiate into T helper cells with a substantial transcriptional heterogeneity. This heterogeneity is not only characterized by differences in effector function and survival but also migration. T-box TF coordinate the expression of several chemokine receptors and thus might have a great impact on trafficking of T cells (29). For example, Eomes regulates the expression of CXCR4, which guides extrafollicular migration of lymphocytes in the spleen and lymph nodes as well as homing to the bone marrow (30). Overexpression of T-bet in CD8^+^ T cells results in their accumulation in the red pulp of the spleen, which is in line with the migration of T-bet dependent KLRG1^high^ CD8^+^ T cells into non-lymphoid peripheral tissue (31). After an acute infection with LCMV, the regular architecture of the spleen is disrupted as antiviral cytotoxic CD8^+^ T cells destroy T cell zone stromal cells (32). Immunofluorescence (IF) microscopy of spleens at day 8 after LCMV WE infection (**Figure 2A**) shows B cell clusters (**Figure 3A**). In addition, we analysed the number and distribution of adoptively transferred SMARTA (CD4^+^CD90.1^+^) and endogenous CD4^+^CD90.1^-^ T cells (**Figures 3A, B**). Corroborating our flow cytometry data we detected a significant reduction in *Tbx21^+/-^* SMARTA CD4^+^ T cells in the spleen. Across all genotypes there was no significant difference in the distribution of SMARTA CD4^+^ T cells. SMARTA CD4^+^ T cells were more abundant outside the B cell zones. In summary, loss of a *Tbx21* allele or timely induction of Eomes in activated CD4 T cells has no profound effect on the distribution during the early phase of an acute infection.

Differentiated CD4^+^ T cells can be identified based on surface markers and effector cytokines. During LCMV WE infection Th1 cells are readily induced through the actions of T-bet, which is promoting canonical Th1 effector functions like IFN-γ production and represses alternative differentiation programs such as Th2 and Th17 (33, 34). Accordingly, flow cytometry analysis of SMARTA *Tbx21^+/+^* CD4^+^ T cells showed strong expression of IFN-γ (**Figure 3C**, upper row), but repression of IL-17 and IL-2 (**Figure 3C**). Haploinsufficiency for *Tbx21* had no effect on IFN-γ production, but *Tbx21^+/-^* CD4^+^ T cells produced less of the cytotoxic effector molecule granzyme B (GzmB). Importantly, we observed an impaired repression of alternative fates with the loss of one *Tbx21* allele, as *Tbx21^+/-^* CD4^+^ T cells produced significantly larger amounts of IL-17 and IL-2 compared to the other two genotypes. Here, Eomes could reverse this de-repression, as *Tbx21^+/E^* CD4^+^ T cells, similar to *Tbx21^+/+^* CD4^+^ T cells, produced hardly any IL-17 and IL-2. In addition, *Tbx21^+/E^* CD4^+^ T cells showed the unique ability of IL-10 secretion, which was therefore under the direct transcriptional control of the *Tbx21^E^* allele (**Figure 3C**, upper row). Next, we explored the expression of surface markers with a special focus on proteins that are linked to Th1 and Tr1 cells (**Figure 3D**). As an internal control we used endogenous non-SMARTA CD4^+^ T cells (**Figure 3D**). In line with our *in vitro* data (**Figure 1E**) T-bet was important for the induction of CD122 and repression of CD127, yet these actions could also be executed by Eomes (**Figure 1D**, upper row). Additionally, Tr1 surface markers like TIM3 and CD27 (10, 25) were more abundant in *Tbx21^+/E^* than in *Tbx21^+/+^* or *Tbx21^+/-^* CD4^+^ T cells (**Figure 3D**). Nevertheless *Tbx21^+/E^* CD4^+^ T cells did not show all markers identifying Tr1 cells as neither CD49b (not shown) was detectable, nor did PD-1 levels differ between the genotypes (**Figure 3D**). Taken together, Eomes and T-bet harbour overlapping functions in inducing Th1 effector functions, while only Eomes induces a Tr1-like phenotype, including IL-10 production.

### Eomes has a profound impact on the transcriptional profile of CD4^+^ T cells

Finally, we wanted to elucidate the impact of Eomes on the transcriptional profile of CD4^+^ T cells by RNA-sequencing (RNA-seq). To do so, we transferred SMARTA CD4^+^ T cells from *Tbx21^+/+^*, *Tbx21^+/-^*, *Tbx21^+/E^* and *Tbx21^-/E^* mice and infected them as shown in **Figure 2**. SMARTA T cells were sorted on day 8 and subjected to RNA-seq. Although Eomes is usually co-expressed with T-bet we added SMARTA *Tbx21^-/E^* mice, to determine whether Eomes alone could govern Tr1 development, which has been questioned in the past (9). Thus, we could evaluate redundant, cooperative, and distinct functions of the two T-box TF in CD4^+^ T cells. For each subset four independent samples were analysed.

**Figure 4:**
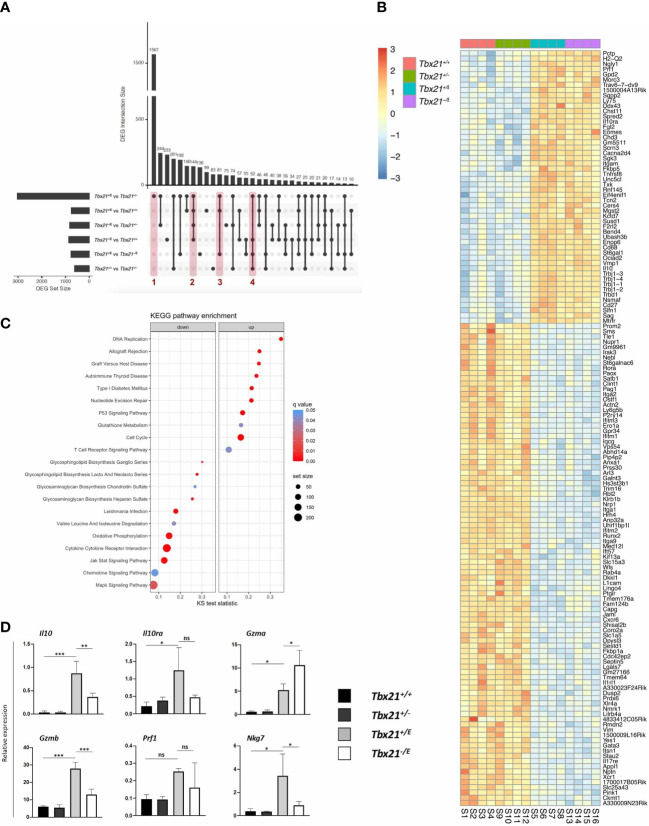
Transcriptional effects of Eomes in CD4^+^ T cells. SMARTA CD4^+^ T cells of the indicated genotypes were transferred into C57BL/6 mice, followed by LCMV infection as shown in **Figure 2A**. On day 8 after infection splenocytes were sorted, RNA isolated and sequencing performed. **(A)** Multi-comparison analysis of all genotypes shown in an upset plot. Set size defines the total amount of differentially expressed genes (DEGs) in this comparison. Intersection size defines the DEGs specifically differentially expressed in the comparisons marked by dots and lines. Red columns mark comparisons further analyzed in (B/Suppl.). **(B)** Heatmap of DEGs marked by column 2 in Figure 4A (*Tbx21^+/E^* and *Tbx21^-/E^* vs *Tbx21^+/+^* and *Tbx21^+/-^*) focussing on the role of Eomes in SMARTA CD4^+^ T cells. **(C)** KEGG pathway enrichment analysis. The x-axis shows the Kolmogorov-Smirnov (KS) test statistic for each pathway, the size of the dots represents the size of each pathway and the colour the statistical significance. **(D)** qPCR of selected targets (IL-10 pathway and cytotoxicity) to validate RNA-seq data from *in vitro* stimulated SMARTA CD4^+^ T cells. Statistical analysis: *=p < 0.05; **=p < 0.01; ***=p < 0.001; ns, not significant; Error bars denote mean + SEM.

Principal component analysis (PCA) clearly showed that the four genotypes clustered separately and were transcriptionally distinct (**Supplementary Figure 1A**). The separation of the groups by principal component 1 (PC1, x-axis) mirrored the number of T-box alleles present in the genotypes, as *Tbx21^+/+^* and *Tbx21^+/E^* (two T-box alleles) diverted from *Tbx21^+/-^* and *Tbx21^-/E^* (one T-box allele). On the other hand, PC2 (y-axis) is best explained by the actions of the *Tbx21^E^* allele, with *Tbx21^+/E^* and *Tbx21^-/E^* separating from *Tbx21^+/+^* and *Tbx21^+/-^*. Taken together, differential expression of T-box TF in our system had a strong effect on variation in gene expression (**Suppl. Figure 1A**). Next, we focussed on the differentially expressed gene (DEG) sets to discern targets and corresponding pathways of T-box TFs. Due to the number of genotypes (*Tbx21^+/+^*, *Tbx21^+/-^*, *Tbx21^+/E^* and *Tbx21^-/E^*), which made Venn diagrams unsuitable, we used upset plots (35) (**Figure 4A**) for multi-comparison analysis. We started by comparing DEG across all four genotypes, which resulted in six sets of DEG with different set sizes (**Figure 4A**, horizontal bars on the left). Next, we identified set intersections between DEG sets (**Figure 4A**, vertical bars), which denotes genes that are similarly regulated in different DEG sets to identify shared pathways. Finally, intersections were ordered according to decreasing size from left to right. In agreement with our PCA analysis, the comparison of CD4^+^ T cells from *Tbx21^+/-^* and *Tbx21^+/E^* showed the highest number of unique DEG (**Figure 4A**, column 1, **Supplementary Table 1**), which were not present in any other comparison. In fact, more than 1500 genes were uniquely differentially expressed between these two genotypes, with about one half up- and one half downregulated. A further analysis of unique DEG intersections allowed us to probe Eomes-dependent changes (**Figure 4A**, column 2, **Supplementary Table 2**), joint actions of T-bet and Eomes (**Figure 4A**, column 3, **Supplementary Table 3**) as well as the effects of two versus one T-box TF allele (**Figure 4A**, column 4, **Supplementary Table 4**). We reasoned that Eomes dependent changes should be present in all DEG intersections that involved either *Tbx21^+/E^* or *Tbx21^-/E^* cells against *Tbx21^+/+^* or *Tbx21^+/-^* cells (**Figure 4A**, column 2). Joint actions of T-bet and Eomes should only be present in intersection of *Tbx21^+/E^* against all other genotypes (**Figure 4A**, column 3). Finally, transcriptional outcome that requires two versus one T-box TF allele should be apparent in intersections of *Tbx21^+/+^* or *Tbx21^+/E^* against *Tbx21^+/-^* or *Tbx21^-/E^* genotypes (**Figure 4A**, column 4). Performing the analysis as such, we identified 144 genes specifically regulated by Eomes (column 2, **Supplementary Table 2**), 81 genes regulated by the joint actions of both T-box TF (column 3, **Supplementary Table 3**) and 52 genes that required two T-box alleles instead of one (column 4, **Supplementary Table 4**) in our data set (**Figure 4A**).

### Eomes is sufficient to regulate a Tr1-like phenotype in CD4^+^ T cells

Next, we focussed on Eomes-specific targets (**Figure 4A**, column 2) in our data. As expected and in support of our mouse model, *Eomes* was uniquely enriched in the DEG intersection of *Tbx21^+/E^* and *Tbx21^-/E^* genotypes (**Figure 4B**). Most interestingly, several Tr1 lineage marker genes were upregulated, including *Il10*, *Il10ra* and *CD27*. The fact, that these genes were also upregulated in the absence of T-bet (*Tbx21^-/E^*) clearly confirms that T-bet is not required for their induction. Intriguingly, the Tr1-associated surface marker CD49b, encoded by *Itga2*, was downregulated in Eomes-expressing genotypes. Besides IL-10 production and signalling, Eomes was also associated with cytotoxic effector molecules (5) as demonstrated by the higher expression of *Prf1* (encodes for perforin). Finally, we also found evidence that Eomes can suppress alternative T cell fates by the repression of Th2 lineage-defining TF *Gata3* in Eomes-expressing genotypes (**Figure 4B**). We next performed pathway analysis to put our DEGs into a broader biological context. Eomes-expressing genotypes showed a significant upregulation in pathways associated with cell cycle and DNA replication as well as several immune-mediated disease that are associated with prolonged antigen-persistence, *e.g.* allograft rejection, Graft versus Host Disease (**Figure 4C**). In contrast, Eomes downregulated oxidative phosphorylation and MAPK signalling, as well as pathways of biomass generation, chemotaxis and cytokine responses (**Figure 4C**). In summary, this set of data would be in line with a model in which Eomes regulates the transcriptional profile of CD4^+^ T cells that evolve during persistent antigen stimulation.

After the analysis of Eomes’ unique functions we next explored the joint actions of T-bet and Eomes, as these are concomitantly expressed in Tr1 cells (**Supplementary Figure 1B**) (9). As only *Tbx21^+/E^* expresses both T-box TF, this genotype was compared with all other genotypes (**Figure 4B**, column 3). Among the 81 DEGs not significantly altered in other comparisons, we found clear evidence that the joint expression of T-bet and Eomes regulates a cytotoxic effector profile. We observed an elevated expression of the cytotoxicity related genes *Ccl5* and *Nkg7*, which encodes for the natural killer cell granule protein-7. This plays an important role in cytotoxic granule release and inflammation (**Supplementary Figure 1B**) (5, 36). Next, we validated our data *in vitro* by stimulating SMARTA CD4^+^ T cells from the four different genotypes and analysed them by flow cytometry and RT-qPCR. Intriguingly, we could demonstrate that Eomes expressing genotypes (*Tbx21^+/E^, Tbx21^-/E^*) showed the highest expression of IL-10 and GzmB, which were co-expressed in the same cell (**Supplementary Figure 1C**). This clearly implicates that Eomes jointly regulates the immunoregulatory molecule IL-10 und and the cytotoxic molecule GzmB. Transcriptional profiling of selected targets corroborated this and could also show that *Il10ra*, *Gzma*, *Prf1* and *Nkg7* were enriched in SMARTA T cells from *Tbx21^+/E^* and *Tbx21^-/E^* mice (**Figure 4D**).

One caveat of our approach using DEG intersections is that it misses stepwise and significant changes in gene expression between *Tbx21^+/E^* and *Tbx21^-/E^* genotypes, as this would result in no intersection. Hence, we also visualized our gene expression data in a heatmap and clustered genes based on Eomes-expressing genotypes (**Supplementary Figure 2**). This approach confirmed our previous analysis, but also showed that the granule-secreted, pro-apoptotic protease Granzyme K (encoded by *Gzmk*) (10) and the Th1-associated chemokine receptor CCR5 (encoded by *Ccr5*) (37) were induced by Eomes (*Tbx21^+/E^* > *Tbx21^-/E^* > *Tbx21* ^+^ *^/^* ^+^ *, Tbx21* ^+^ *^/-^*) and showed the highest expression levels in *Tbx21* ^+^ *^/E^* CD4^+^ T cells. In contrast, Eomes showed a strong effect on alternative fates, as lineage-defining TFs *Rorc*, including downstream targets *Il17a* and *Ccr6*, and *Gata-3* were strongly repressed in Eomes-expressing CD4^+^ T cells (**Supplementary Figure 2**).

**Figure 5:**
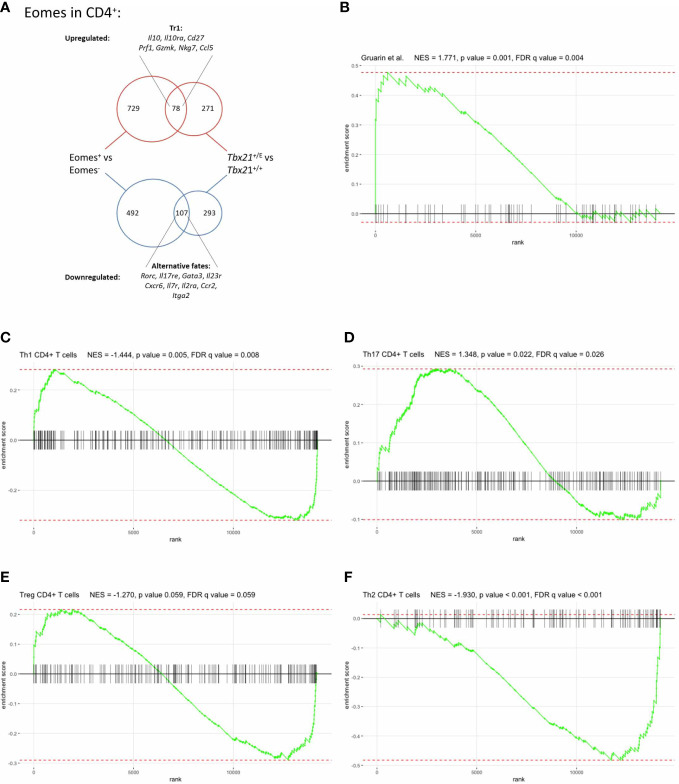
Eomes governs a distinct transcriptional profile in CD4^+^ T cells. **(A)** Comparison of Eomes-specific gene set with Roessner et al. (11) DEGs of *Tbx21^+/+^* vs *Tbx21^+/E^* were compared with the DEGs of Roessner et al. Eomes^+^ vs Eomes^-^ CD4^+^ T cells after expansion in *Rag2^-/-^* mice. Of the 78 up- and 107 downregulated genes differentially expressed in both studies selected genes are shown (Full list in **Supplementary Table 5**). **(B-E)** GSEA of T helper cell subset gene signatures compared with differences in gene expression between CD4^+^ SMARTA T cells from *Tbx21^+/E^* and *Tbx21^+/+^*, **(B)** a set of genes upregulated in human IL-10-secreting IL-7r^-^ CD4^+^ T cells, derived from Gruarin et al. (10), **(C-F)** sets of upregulated genes in **(C)** Th1, **(D)** Th17, **(E)** Treg and **(F)** Th2 CD4^+^ T cells, as detailed in the text. The enrichment score (ES) is shown on the y-axis and the x-axis represents the sorted list of genes in our comparison. Genes that are present in the respective gene set are shown by vertical black lines.

To externally validate our model and results we next compared our Eomes-specific transcriptome with another mouse model. Recently, Roessner et al. utilized *Eomes^Gfp/+^*-reporter mice (38) to compare the transcriptional profile of Eomes^+^ and Eomes^-^ CD4^+^ T cells after transfer into *Rag2^-/-^* mice and homeostatic expansion three weeks later (11). We analysed their published RNA-seq data and identified DEG between Eomes^+^ and Eomes^-^ CD4^+^ T cells. This external DEG set was compared to DEG between *Tbx21^+/E^* and *Tbx21^+/+^* CD4^+^ T cells from our study, as these two comparisons focussed on the role of Eomes in CD4^+^ T cells and thus were comparable. The DEG intersection consisted of 187 similarly regulated genes between both comparisons, 78 were upregulated and 107 downregulated (**Figure 5A**; table 5) in both DEG sets. Tr1-specific genes like *Il10*, *Il10ra*, *Cd27*, *Prf1*, *Gzmk*, *Nkg7* and *Ccl5* were clearly upregulated in both DEG sets. Hence, the immunoregulatory cytokine IL-10 and cytotoxic effector functions are both regulated by Eomes in Tr1 CD4^+^ T cells. The overlap of downregulated DEGs included genes that suppressed alternative CD4^+^ T cell fates, like *Rorc* or *Gata3*, further confirming the lineage-defining role of Eomes for Tr1 CD4^+^ T cells (**Figure 5A**; **Supplementary Table 5**).

### Eomes-driven transciptome in CD4^+^ T cells is distinct from other conventional T helper cell signatures

Finally, we investigated whether the identified gene set of *Tbx21* ^+^ *^/E^* CD4^+^ T cells is unique and sufficient to discriminate Eomes-expressing CD4^+^ T cells from other T helper cell lineages. Due to the limitations of comparing individual genes we applied Gene Set Enrichment Analysis (GSEA) with published data sets for Tr1 T cells and conventional CD4 T helper cell subtypes. Intriguingly, gene sets for IL-10 producing CD4^+^ T cells (10) were significantly enriched in *Tbx21* ^+^ *^/E^* CD4^+^ T cells (**Figure 5B**), which validated our reverse genetic approach. In contrast, gene sets for murine Th1 (**Figure 5C**), Th17 (**Figure 5D**) and Treg (**Figure 5E**) CD4^+^ T cells (20) displayed a bimodal enrichment pattern, as there is a large accordance with these gene sets, but also a large complementarity. Finally, GSEA for Th2 gene sets showed a negative enrichment score implicating that genes that are down-regulated in Eomes-expressing CD4^+^ T cells are up-regulated in Th2 (**Figure 5F**) CD4^+^ T cells, as demonstrated for GATA-3 (**Figure 4B** and **Supplementary Fig 2**).

In summary, our in-depth transcriptome analysis, including several internal controls and external validation, revealed a specific transcriptional program in CD4^+^ T cells driven by Eomes. This program includes the induction of the immunoregulatory cytokine IL-10 as well as cytotoxic effector functions.

## Discussion

Eomes expression in CD4^+^ T cells has been found in mouse models of chronic inflammation and inflammatory disorders in human, but its specific functions remain astonishingly unclear. Several non-exclusive roles have been proposed for Eomes in CD4^+^ T cells as it may promote inflammation and tissue damage through inflammatory cytokines, inhibit tumor growth through cytotoxic effector functions and ameliorate inflammation through the elimination of antigen-presenting cells and the release of IL-10 (6–8, 11). Similar observations have been made in humans and it therefore appears that ultimately, transcriptional actions of Eomes^+^ CD4^+^ T cells will be context-dependent and modified by additional factors (4).

In contrast to CD8^+^ T cells, where Eomes is readily found and induced during an acute infection, Eomes induction in CD4^+^ T cells requires particular models, which has hampered a thorough understanding of its role. Our model takes advantage of this fact, as we show here that Eomes is not induced in CD4^+^ T cells during the acute phase after LCMV WE infection and therefore all effects observed in T cells carrying the *Tbx21^E^* allele can be ascribed to the actions of the *Tbx21^E^* allele. We show that in the *Tbx21^E^* mouse model Eomes is readily induced in CD4^+^ T cells under Th1 priming conditions *in vitro* and *in vivo* and mirrored the expression dynamics of T-bet from the *Tbx21^+^* wild type allele. In contrast, Eomes expression in wild type CD4^+^ T cells occurs late (> day 30) during a chronic infection and the transcriptome of such CD4^+^ T cells differs strongly from CD4^+^ T cells during an acute infection (13). Hence, our model is not suited to predict the complex transcriptional profile of Eomes^+^ CD4^+^ T cells during a chronic infection. Nevertheless, we were able to define the core profile of Eomes in CD4^+^ T cells by decoupling its induction from extrinsic cues in a unique genetic model (21). In fact, we show here that Eomes regulates important aspects of Tr1 differentiation like IL-10 production and signalling, but also displays functional overlap with T-bet pertaining to the control of cytotoxic effector functions and repression of alternative CD4^+^ T cell fates. So far, Tr1 CD4^+^ T cells are by and large defined by their ability to produce the immunoregulatory cytokine IL-10. However, IL-10 can be produced by a variety of haematopoietic cells (39), including myeloid and lymphoid cells, which makes its sole use unreliable for identifying Tr1 CD4^+^ T cells. Recently, Zhang et al. identified Eomes as a lineage-specifying transcription factor for Tr1 T cells, however, they also reported that *in vitro* induction of IL-10 in CD4^+^ T cells with IL-27 and TGF- β did not induce Eomes (9). Moreover, co-expression of CD49b and LAG-3 (25) cannot be used to trace Eomes-expressing Tr1 T cells in the context of bone marrow transplantation (9) and we also did not discern these markers in our study. Finally, multiple transcription factors promote IL-10 production in Tr1 cells (9, 40–42) and it is likely that Eomes will not be the only TF that regulates IL-10 in CD4^+^ T cells. In summary, IL-10 producing CD4^+^ T cells display a substantial heterogeneity, and it will have to be determined in the future whether these are context-dependent *bona fide* subsets or rather developmental stages.

As expected from the phenotypical data, RNA-seq revealed a distinct transcriptional program controlled by Eomes. Interestingly, the CD4^+^ T cell transcriptome was altered by the type, as well as by the allele frequency of T-box TF, implicating their non-redundant and finely tuned regulation. As a result, we were able to outline specific effects of Eomes, common targets with T-bet and cooperative effects of both T-box TF. Even in the absence Tr1-forming conditions Eomes expression was sufficient to induce Tr1-associated transcriptional changes, including *Il10*, *Il10r* and *Cd27*, which support its role as a lineage-specifying TF. In addition, we identified a cytotoxic effector profile, including *Prf1*, *Gzmb*, *Gzmk*, *Nkg7* and *Ccl5* (5), which was most pronounced when CD4^+^ T cells expressed T-bet and Eomes. This is in line with the ability of cytotoxic Eomes^+^ CD4 T cells to aggravate autoimmune disease but also to contribute to viral clearance and elimination of malignant cells. Mechanistically, killing of target cells is assumed to be mainly regulated by contact dependent MHC class II-restricted antigen recognition, which triggers secretion of the pore-forming protein perforin 1 together with apoptosis-inducing serine proteases, called granzymes (5). Hence, dysregulation of cytotoxicity can lead to severe tissue damage and aggravate autoimmunity. Raveney et al. found Eomes^+^ CD4^+^ T cells in patients with secondary progressive multiple sclerosis and in the corresponding EAE mouse model. A potential neurotoxic mechanism dependent on GzmB correlated with expression of Eomes (6, 7). On the other hand, cytotoxic Eomes^+^ CD4^+^ T cells accumulate during chronic infections in mice and humans (43, 44), where they might compensate for an exhaustion-induced loss of cytotoxicity from CD8^+^ T cells (45) or virus-induced downregulation of MHC class I molecules. Additionally, Eomes^+^ CD4 T cells might mitigate overt inflammation during chronic infection by recognizing and eliminating MHC class II^+^ antigen-presenting cells. Finally, cytotoxic Eomes^+^ CD4 T cells can mediate anti-tumor activity as shown in models of chronic lymphocytic leukemia (CLL) (11). Intriguingly, in this latter study transcriptome analysis of CLL-induced Eomes^+^ CD4 T cells showed a striking overlap with our own analysis and corroborates our conclusion that Eomes regulates both, immunoregulatory IL-10 secretion and cytotoxic effector functions. Regulation of cytotoxic functions of CD4^+^ T cells may therefore be of special interest in tumor therapy or chronic infections with T cell-based approaches (11). Finally, we also demonstrated that the lineage-specifying actions of Eomes for Tr1 differentiation are established and reinforced through repression of alternative T cell fates, as *Gata3* and *Rorc* were significantly downregulated. This might explain why Eomes expression can lead to the plasticity of Th17 cells by instructing their trans-differentiation towards Th1 T cells and aggravating colitis (8) in a T cell transfer model.

In summary Eomes drives a distinct transcriptional and phenotypical program in CD4^+^ T cells. This program includes, but is not limited to, the induction of the immunoregulatory cytokine Il-10, a cytotoxic effector profile and repression of alternative fates. It may therefore not only act as a lineage-specifying TF in Tr1 differentiation, but also influence CD4^+^ T cell plasticity depending on the transcriptional context, potentiating or limiting pro-inflammatory mechanisms. Deciphering the molecular pathways of Eomes may lead to the identification of new therapeutic targets to ameliorate states of chronic inflammation or malignant disease.

## Data availability statement

The datasets presented in this study can be found in online repositories. The names of the repository/repositories and accession number(s) can be found in the article/**Supplementary Material**.

## Ethics statement

The animal study was reviewed and approved by Animal care committees of the University of Freiburg and the Regierungspräsidium Freiburg.

## Author contributions

BT, PK, JH, FA and LK performed experiments. VS performed sequencing analysis. BT, VS, MB and YT analyzed and interpreted the data. JH, CK, SJA and YT generated the *Tbx21^E^* mouse model. BT and YT wrote the manuscript. YT supervised the study and revised the manuscript. All authors contributed to the article and approved the submitted version.
