# Supplementary figures and images for: Eomes is sufficient to regulate IL-10 expression and cytotoxic effector molecules in murine CD4+ T cells
Source: Front Immunol. 2023 Jan 19;14:1058267. doi: 10.3389/fimmu.2023.1058267 (PMC9901365; doi:10.3389/fimmu.2023.1058267)

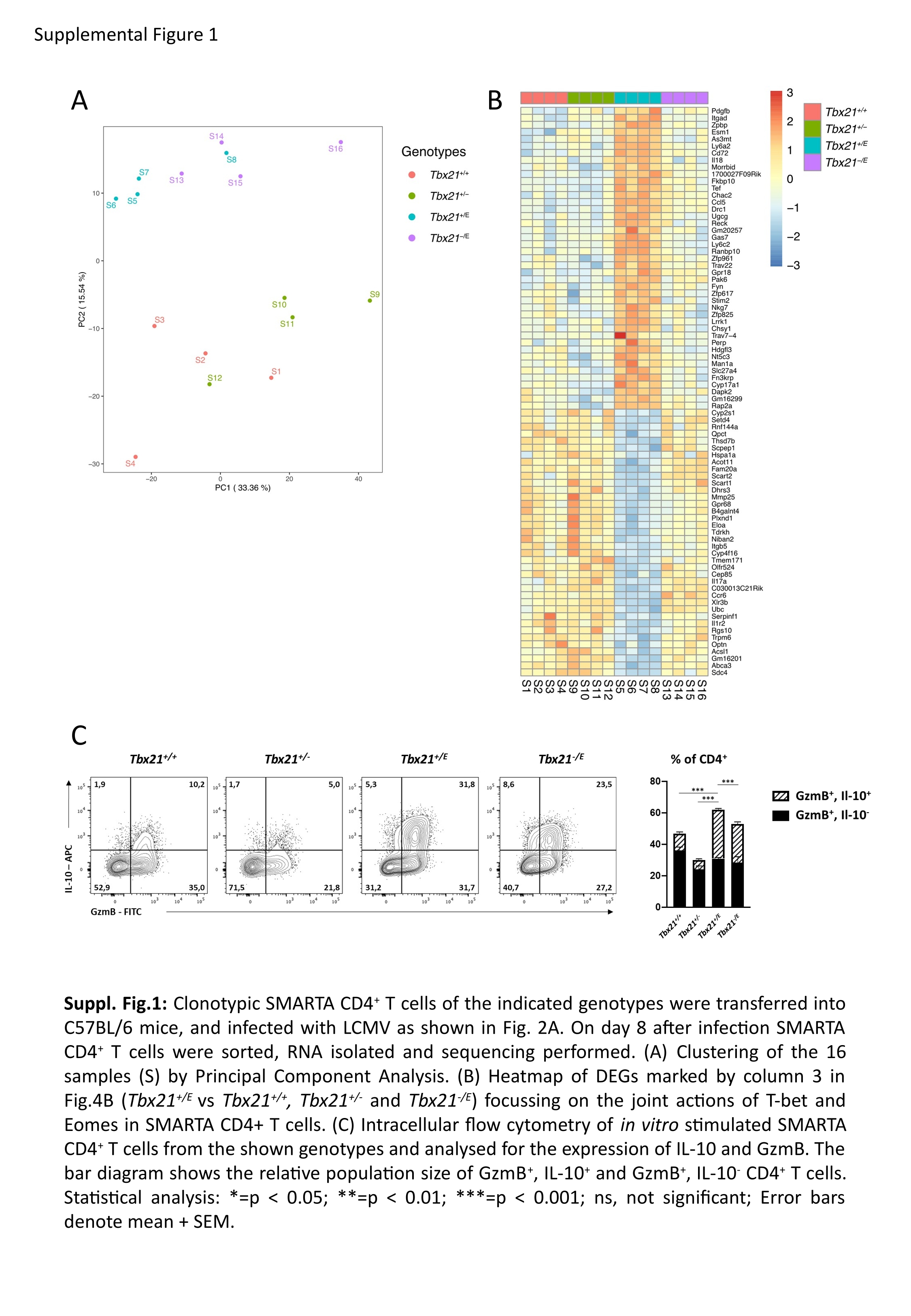

Supplement: Supplementary file 1 [file Image_1.jpeg]

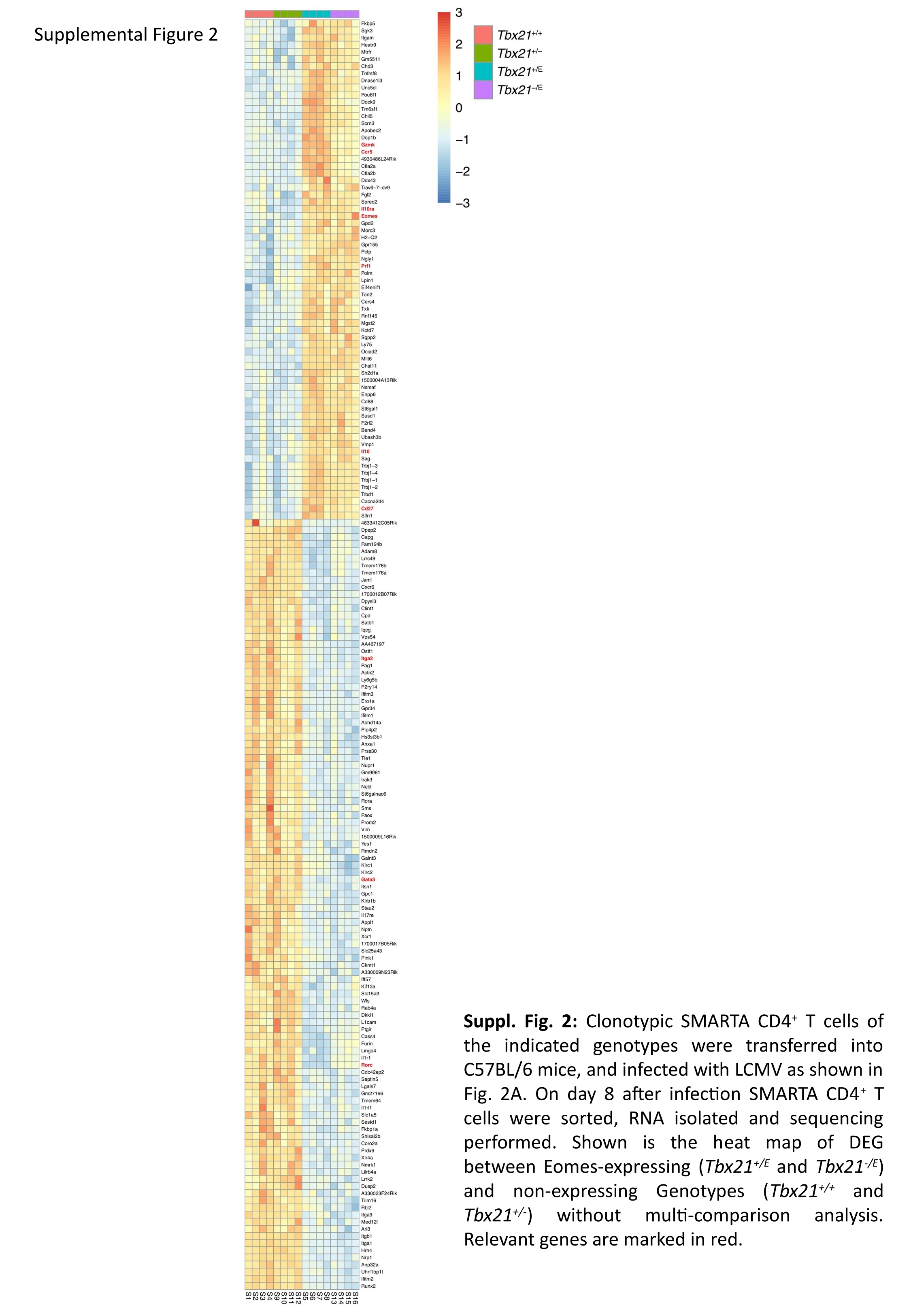

Supplement: Supplementary file 2 [file Image_2.jpeg]
